# Both Rewards and Moral Praise Can Increase the Prosocial Decisions: Revealed in a Modified Ultimatum Game Task

**DOI:** 10.3389/fpsyg.2018.01865

**Published:** 2018-10-04

**Authors:** Xiangling Wang, Jiahui Han, Fuhong Li, Bihua Cao

**Affiliations:** ^1^School of Psychology, Jiangxi Normal University, Nanchang, China; ^2^College of Teachers, Chengdu University, Chengdu, China

**Keywords:** fair, redivision, social norms, compensation, moral praise

## Abstract

Unlike other creatures, humans developed the ability to cooperate with genetically unrelated strangers and a tendency to comply with social norms. However, humans deviate from social norms in various situations. This study used the modified ultimatum game to explore why humans deviate from social norms and how their prosocial behavior can be promoted. In Study 1, participants were asked to imagine working with an anonymous counterpart to complete a task and obtain a certain amount of money (e.g., ¥10). The computer divided the money randomly in favor of the participant (e.g., 9:1 or 8:2). Participants should decide whether to accept or reject such a self-benefiting division. In the non-risk condition, an absolutely fair redivision of money would take place if participants reject self-benefiting division (e.g., 5:5 or 6:4). By contrast, in the risk condition, other-benefiting redivision of money (e.g., 1:9 or 2:8) would take place if participants rejected the self-benefiting division. Results involving 40 college students showed the main effect of condition. The frequency of accepting self-benefiting division in the risk condition was higher than that in the non-risk condition. As such, compliance with social norms is based on the preservation of material resources. In Study 2, we used economic or moral rewards to compensate for economic loss following compliance with the norm. Results involving 28 college students revealed a significant effect of compensation. The rewards, including moral praise, effectively decreased selfish choices. These findings extend previous studies on social norm compliance by emphasizing the importance of internal, fairness-based balance between material and moral needs, as well as the role of moral praise in promoting prosocial behavior.

## Introduction

Rules of justice originate from regularities in behavior in relation to mutual interactions among humans (Hume, 1978). Convention refers to a general expectation of conformity to a particular regularity that everyone is interested in complying with (Sugden, 1986, 1998). Most of us resent individuals whose behaviors contradict expectations and conventions. Moreover, the majority of people feel uneasy when they become the focus of resentment of others; their desire to avoid resentment can improve prosocial actions (Sugden, 1998; Fehr and Gächter, 2000). These social cognitions and emotions are related to the nature of social norms.

### Nature of Social Norms

Expectation is one of the main ingredients of the norm (Sugden, 1986; Lewis, 2008; Bicchieri and Xiao, 2009). According to Lewis (2008), every member of the society expects others to conform; everyone will actually conform if everyone else conforms to conventions. Bicchieri (2005) argued that obedience to the norms depends on empirical and normative expectations. Normative expectations refer to what individuals believe others think ought to be done, whereas empirical expectations refer to what individuals expect others to do (Bicchieri, 2005).

For example, when two or more persons participate in a task, each person expects everyone to exert their best effort to finish the work. In addition, each person will likely expect that everyone should receive a fair distribution of the total outcome or rewards of their hard work. The former expectation forms cooperation, which is ubiquitous in biological systems (Nowak and Sigmund, 2004; Doebelli and Hauert, 2005), whereas the latter forms fairness-based norms. Cooperation is observed in many levels of social groups; it is the decisive organizing principle of human society from primitive tribes to modern nation states (Nowak, 2006; Ghang and Nowak, 2015).

Studies that utilized different versions of ultimatum game (UG) demonstrated that the majority of people demand fairness and are willing to punish unfair behavior at a personal cost (Radke et al., 2012; Yu et al., 2015). In UG, two players, a proposer and a responder, work together to split a certain amount of money. The proposer can transfer any amount of money, while the responder can either accept or reject the offer. If the offer is accepted, both players receive the corresponding money. Otherwise, neither of them receives money. According to standard game theories, a completely rational proposer would transfer the minimal offer, and the responder would accept any offer larger than zero given that the offer is better than nothing. However, previous studies showed that proposers typically offer about 40% of the total money; offers below 20% are rejected by responders (Güth et al., 1982; Camerer, 2003). These findings imply the proposer and the responder induce normative expectation on fairness in splitting the money. Under this expectation, the proposer will comply voluntarily with the social norm of fairness in money division. Bicchieri and Chavez (2010) used a modified UG and found that adults conform to the principle of fairness only when the convention-based institution^1^ is built soundly. However, when the institution is not well structured, individuals possibly think receivers would not know even if they deviate against fairness, and thus, would not reject unfair division. Therefore, the probability of proposing an unfair division is substantially increased.

In brief, the nature of social norms seems to be based on common economic interests of all individuals, with common expectations, conformity, and mutual knowledge of their action as premises. However, an individual might be confused when confronting the conflict or dilemma among his (or her) interests, those of others, immediate and long-term interests, and economic interests and moral reputation.

### Social Dilemmas in Mutual Interaction

Many societal problems are caused by a conflict between one’s and others’ benefits. These conflicts are called social dilemmas (Dawes, 1980; Irwin et al., 2014). A long-standing tradition in economic models views human beings as exclusively self-interested; they tend to become less cooperative in social dilemmas when money is involved (Fehr and Gächter, 2000; Kouchaki et al., 2013). According to evolutionary theories, survival is based on fierce competition among individuals; thus, natural selection favors defection and selfish behavior if no mechanism exists for the evolution of cooperation (Nowak, 2006; Dreber et al., 2008; Grimalda et al., 2016). Cooperators are always more fit than defectors (Nowak, 2006). Cooperation may have been the main survival strategy of humans, and it would have been selected for adaptations (Brewer and Caporael, 1990; Buss, 1995). However, numerous individuals might be uncooperative or deviate from group norms in particular circumstances.

To enable individuals to constrain their selfish behavior while showing prosocial behavior, social norms should constitute standards of behavior for how individuals should behave in a given situation, particularly when confronting social dilemma (Elster, 1989; Spitzer et al., 2007; Ruff et al., 2013; House, 2018). Everyone should step out of the social dilemma and comply with social norms. Researchers identified different types of compliance with social norms, namely, voluntary, emotion-based, and sanction-induced compliance (Fehr and Urs, 2004; Ruff et al., 2013). Many studies have highlighted the importance of sanctioning violators (Fehr and Gächter, 2002; Fehr and Fischbacher, 2003; De Quervain et al., 2004; Spitzer et al., 2007; Houser et al., 2008; Ruff et al., 2013). Violation of social norms challenges the existence of norms and moral values within the community; sanction is a means of reasserting and defending these norms and moral values (Darley and Pittman, 2003). In UG, responders might incur costs to punish norm violators and enforce fairness (Fehr and Gächter, 2002).

The importance of credibly sanctioning threats to maintain compliance of norms and moral values is well established by ethnographic evidence, evolutionary theory, and laboratory studies. However, little attention has been paid to why people deviate from social norms and how prosocial behavior can be promoted. It has been suggested that repeated interactions can induce possible strategies in determining whether to cooperate or deviate in the next round based on the outcome of earlier rounds (Doebelli and Hauert, 2005).

Bicchieri and Xiao (2009) demonstrated that manipulation of expectations would effectively change people’s conformity to social norms. They used a modified Dictator Game (DG), which has been widely adopted to study fairness (Bardsley, 2007; Bicchieri and Xiao, 2009; Korenok et al., 2017). In the standard version of DG, two subjects are paired randomly, one as dictator or proposer and the other as a receiver. The dictator proposes how much of $10 he or she wants to send to the receiver. Bicchieri and Xiao (2009) designed a conflict between normative and empirical expectations. In the empirical expectation condition, they presented each dictator with a message that summarizes the majority of the dictators’ actual choices in one of the previous sessions. After making a decision, the experimenter asked each dictator how many dictators they believed split the money equally in the current session. In the normative expectation condition, the researchers presented each dictator with a message that summarizes what the majority believe should be done (e.g., 60% of dividers who participated in a session of this experiment mentioned that dividers should share the amount equally). After making the decision, the experimenter first asked each dictator whether they thought dictators should split the money equally. They were then asked how many dictators they believed answered “yes” to the first question. The results indicated that empirical expectations about other dictators’ behaviors and not normative expectations are the key factors that influence dictators’ choices.

In brief, when confronting social dilemmas, people should behave prosocially even if they instinctively want to maximize their own benefits. Compliance with social norms is voluntary, emotion-based, sanction induced, or expectation driven.

### Objectives of This Study

Previous studies confirmed that individuals behave with fair prosocial motivations; however, in reality, people often perform unethical behaviors, such as lying, deception, cheating, stealing, sabotaging, or breaking the law (Feldman et al., 2015). The prevalence of unethical behavior and the fact that even good people are prone to lose track of their moral compass is surprising (Mazar et al., 2008).

Why do prosocial individuals violate social norms at the cost of sacrificing moral pursuits? Existing studies have demonstrated that individuals’ empirical expectant and the repeat interaction between individual and environment can change prosocial behavior (Doebelli and Hauert, 2005; Bicchieri and Xiao, 2009). Accordingly, we hypothesized that individuals will deviate from social norms due to the repeated negative outcome of their prosocial behavior (e.g., their economic incomes are unexpectedly reduced after compliance). Thus, individuals initially have prosocial motivations. However, they will be compelled to withdraw moral pursuits on subsequent actions for protecting their material resources when they receive repeated loss of material resources due to compliance with social norms.

To test this hypothesis, we used a modified UG and controlled the degree of fairness in redivision of total money (Study 1). If participants comply with social norms – that is, they reject the self-benefiting (e.g., 8:2) – they would absolutely receive fair redivision of money (e.g., 5:5) in the non-risk condition. However, in the risk condition, they could possibly receive an unfair redivision of money (e.g., 3:7) – that is, they might receive even less money than they deserved. In Study 1, we predicted that participants in the risk condition might deviate from the social norm. Hence, they might accept the self-benefiting division more frequently than they did in the non-risk condition. By contrast, they will receive fair redivision of money after they reject the self-favorable offer under non-risk condition. Thus, their economic interests were not reduced when they adhered to the principle of fairness. Therefore, they will continue to exhibit norm conformity in subsequent trials. The result of Study 1 is important to establish the experimental paradigm of Study 2.

In Study 2, we identify how people who deviate from social norms can be guided to comply with social norms once more. Social scientists suggest that the sanctioning system effectively solves social order issues (Fehr and Gächter, 2002; Fehr and Fischbacher, 2003; Houser et al., 2008). However, numerous studies demonstrated that sanction is costly and inefficient, thereby resulting in considerable monetary losses (Amor and Fort, 2011) or destructive acts of vengeance (Nikiforakis, 2008). In addition, we argued that sanctions as a traditional way to maintain social norms become counterproductive when used to punish the violator deviating from social norms due to loss of his (or her) earned material resources following repeated compliance. Complying with social norms but suffering material loss lacks fairness, thereby prompting individuals to violate the norms on subsequent actions. Punishment for this type of defection would form a stereotypical view that earned material resources would be lost regardless of compliance with social norms. Thus, the internal prosocial motives and fair consideration of individuals would be destroyed.

In comparison with sanctions, rewards evoke a higher level of cooperation (Parks, 2000; Wang et al., 2014). According to Sanfey et al. (2014), investigators should explore how social and monetary incentives might differently influence social norm compliance. Previous studies only used economic rewards, whereas moral rewards have not been used to date. The individual desire to establish positive social image is a more decisive factor in promoting human cooperation than punishment (Grimalda et al., 2016). Moreover, moral sentiments play an important role in human decisions that can go beyond the maximization of material gain (Amor and Fort, 2011). Accordingly, we predict that the individuals in Study 2 would comply continuously with social norms if they receive moral or economic compensation.

## Study 1

### Method

#### Participants

We recruited 40 (20 males with mean age 23 ± 1.69 years old) undergraduate and graduate students from Liaoning Normal University in China. No significant gender difference was observed in any measure. After entering the laboratory, participants were asked to engage in the UG task. Participants were informed they would be paid ¥20 as basic payment after the experiment. Additional monetary rewards were dependent on their total earnings in the UG task by a certain ratio. For example, if a participant earned ¥30,000 in the task, then the additional reward is 30000 × 0.001 = ¥30. Hence, the final pay for participation is 20 + 30 = ¥50. The written informed consents of each participant were collected. The study was approved by the local research ethics committee of Liaoning Normal University.

#### Design and Materials

A 3 (total amount: ¥100, ¥1,000 and ¥10,000) × 4 (division scheme: 9:1, 8:2, 7:3 and 6:4) × 2 (condition: risk, non-risk) within-participant design was used. The acceptance rate of division schemes was used as dependent variable.

**FIGURE 1:**
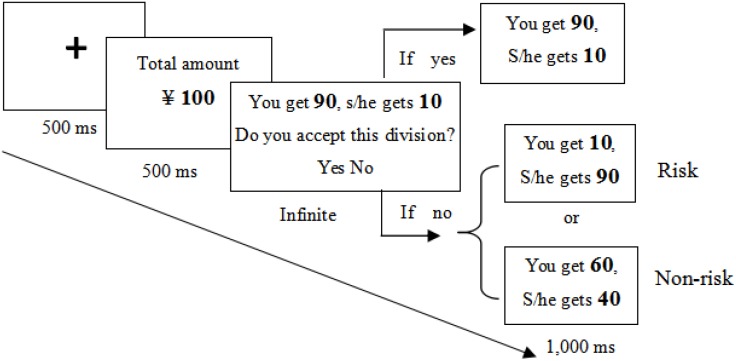
Trial procedure in different conditions in Study 1.

We developed a modified version of UG, wherein the participants were asked to imagine working with a stranger to complete a task and obtain a certain amount of money (e.g., ¥10). Their contributions were identical, and both deserved half of the remuneration. However, the computer randomly divided the money in favor of the participant (9:1 and 8:2 as extremely self-benefiting division schemes because the participant receives 90 or 80%, whereas the counterpart obtains 10 or 20% of the total remuneration; 7:3 and 6:4 as moderately self-benefiting division schemes). Participants should decide whether to accept or reject the self-benefiting division. If they accepted, the money was divided accordingly, and participants would receive a larger amount of money than their counterpart. Otherwise, the money was redivided (**Figure 1**).

Each participant performed two conditions (blocks) of the game. In the non-risk block, if participants rejected the random division that is self-benefiting but unfair to the counterpart, then they always received a positive outcome (i.e., a fair redivision). Thus, they would get 40, 50, or 60% of the total remuneration. We provided unfixed rather than fixed remuneration (e.g., 5:5) to elicit the uncertainty of redivision. In the risk block, if participants rejected, then they possibly received a negative outcome. Thus, they might merely get 10 or 20% of the total remuneration in the redivision (see **Appendix**). Participants were unaware of the probability of unfair redivision. Each total amount and division scheme was presented 10 times. Thus, each block had 120 trials. Risk and non-risk blocks were presented randomly.

#### Procedure

Upon arrival at the laboratory, each participant was asked to understand the rules of the game by playing with a new counterpart in every trial. They were informed that they will receive a basic payment for participation plus earnings from the game. Participants were asked to provide “acceptance” or “rejection” decision using his (or her) left or right index finger.

Unknown to the participants, the amounts of the total money and division schemes were manipulated by the experimenter and presented in random order. As shown in **Figure 1**, each trial began with a cross-presentation for 500 ms, followed by the presentation of a total amount of money for another 500 ms. A division scheme was then presented infinitely while two options (“Yes” or “No”) remained visible. If participants responded with yes, then the subsequently distributed outcome was displayed on the screen for 1 s. However, if they rejected it, then the outcome (redivision) was then displayed for 1 s.

### Results

**FIGURE 2:**
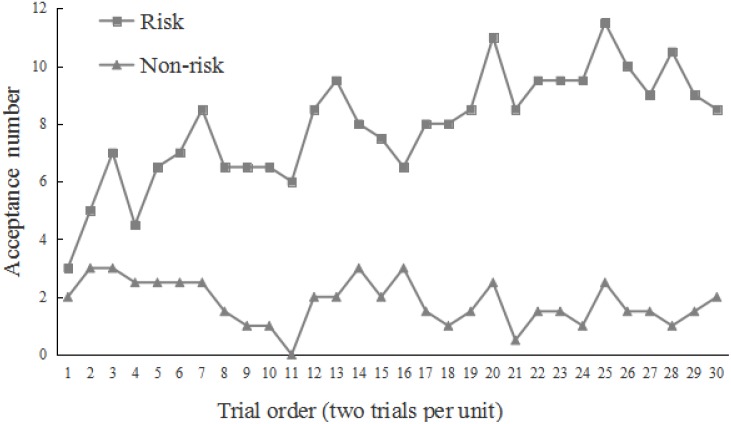
Change of acceptance on extremely self-benefiting division schemes (9:1 and 8:2) in the different conditions. The *y*-axis refers to the number of acceptance for every two trials. For example, if four participants accept the division at the 11th trial and six participants accept the division at the 12th trial, then the mean acceptance for the 11–12th trials is 10.

As shown in **Figure 2**, the number of acceptance^2^ was rather low in the first two trials, regardless of whether the condition was risk or not. However, the acceptance gradually increased in the risk condition.

Preliminary analysis indicated that the data met the assumptions for ANOVA; thus, repeated measures ANOVA was performed on the acceptance rate, with total amount, condition, and division scheme as within-subject factors. Results showed that the main effect of condition was significant, *F*(1,39) = 146.44, *p* < 0.001, η^2^ = 0.79, β = 0.05. The mean acceptance rate in the risk condition (43%) was significantly higher than that observed in the non-risk condition (21%). In addition, the main effect of division scheme was significant, *F*(3,117) = 100.10, *p* < 0.001, η^2^ = 0.72, β = 0.03. Acceptance rate increased as the division scheme neared fairness (5:5). No effect of total amount was observed, *F*(2,78) = 2.78, *p* = 0.07, η^2^ = 0.07.

**FIGURE 3:**
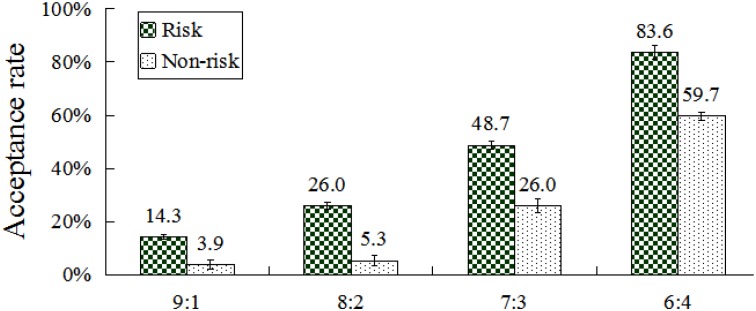
Acceptance rates at different conditions and division schemes. Error bars index SE.

An interaction between condition and division scheme was found, *F*(3,117) = 7.32, *p* < 0.001, η^2^ = 0.16. *Post hoc* pair-wise comparisons (Bonferroni correction) revealed that acceptance rates differed significantly within each pair of division schemes in the risk condition (all *p*s < 0.001). In addition, acceptance rates differed significantly between any pair of division schemes in the no-risk condition (all *p*s < 0.001), except for the pairs of 9:1 and 8:2. For all division schemes, acceptance rates in the risk condition were significantly higher than in the no-risk condition (all *p*s < 0.01) (**Figure 3**).

**FIGURE 4:**
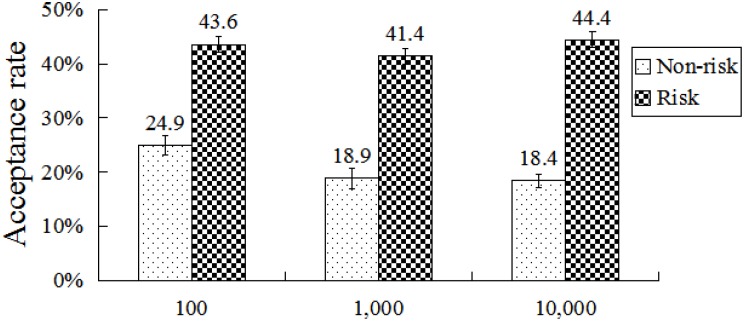
Acceptance rates at different conditions and total amounts. Error bars index SE.

An interaction was observed between total amount and condition, *F*(2,78) = 3.49, *p* < 0.05, η^2^ = 0.08. *Post hoc* analysis revealed that acceptance rates in the risk condition did not differ significantly between any pair of total amounts. However, acceptance rates in the non-risk condition differed significantly between each pair of total amounts (all *p*s < 0.01), with the exception between ¥1,000 and ¥10,000. Acceptance rates in the risk condition were significantly higher than those observed in the non-risk condition with any total amount (all *p*s < 0.01) (**Figure 4**).

**FIGURE 5:**
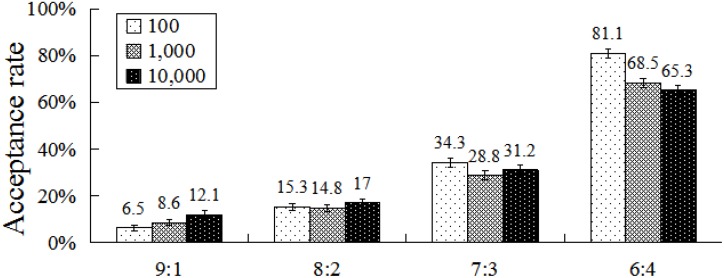
Acceptance rates at different total amounts and division schemes. Error bars index SE.

A significant interaction was observed between total amount and division scheme, *F*(6,234) = 9.21, *p* < 0.001, η^2^ = 0.19. *Post hoc* analysis revealed that acceptance rates differed significantly between any two total amounts for the 9:1 division scheme (all *p*s < 0.05), except for the ¥100 and ¥1,000 pair. For the 8:2 division scheme, acceptance rates did not differ significantly within each pair of total amounts. For the 7:3 division scheme, acceptance rates differed significantly only within the ¥100 and ¥1,000 pair (*p* < 0.05). For the 6:4 division scheme, acceptance rates differed significantly within each pair of total amounts (all *p*s < 0.05), with the exception between ¥1,000 and ¥10,000. In addition, acceptance rates differed significantly within any two division schemes (all *p*s < 0.05), for all total amounts (**Figure 5**).

### Discussion

The results of Study 1 showed that the acceptance rate in the risk condition was significantly higher than that in the non-risk condition. In the non-risk condition, redivision was fair in reserving their earned material resources when participants rejected the self-benefiting but unfair division for counterparts. Consequently, their needs for moral (fairness) pursuits and material resources were met, thereby resulting in continuous prosocial behavior. However, in the risk condition, participants might repeatedly suffer negative outcome (unfair redivision resulting in an ultimate loss in material resources) if they always rejected self-benefiting choice. Thus, participants attained moral pursuits at the cost of sacrificing material resources. Therefore, the internal balance between material and moral pursuits was damaged. To protect their material resources, participants had to forgo fairness and accept self-benefiting options. Therefore, Study 1 demonstrated that the positive at least neutral outcome (i.e., fair-based redivision) for their initial action is the precondition of continuous prosocial behavior rather than the maximization of self-benefit.

The results showed that the acceptance rate of participants increased in the risk condition as the division scheme neared fairness. Moderately self-benefiting divisions (7:3 and 6:4) were more frequently accepted than extremely self-benefiting division schemes (9:1 and 8:2). In the non-risk condition, acceptance rates for 9:1 (3.9%) and 8:2 (5.3%) division schemes were very low and did not differ substantially. If participants accepted extremely self-benefiting divisions, then they would obtain more money than they deserved. However, their counterparts would suffer the larger loss of material resources, which apparently deviate from their fairness consideration. Therefore, they gave up the maximization of self-benefit in the non-risk condition. These findings are consistent with previous studies, wherein participants preferred fairness and were concerned about the interests of others (Radke et al., 2012; Yu et al., 2015).

In addition, the acceptance rate of ¥10000 in the 9:1 division scheme was higher than those of ¥100 and ¥1000. However, for the 6:4 division scheme in the non-risk condition, the acceptance rate of ¥100 was higher than those of ¥1000 and ¥10000. Participant earnings of material resources might suffer a larger loss if they rejected the 9:1 scheme when the amount of total money was ¥10000. Thus, the material resources reservation is the premise of moral pursuits. However, for the 6:4 division scheme, the money difference between self and counterpart was the least obvious when the amount was ¥100. Thus, economic loss is small in redivision. Therefore, the amount of money would influence people’s fair consideration to a certain degree.

In brief, Study 1 revealed that people who initially conformed to the norm could deviate mainly because they repeatedly received negative outcome (i.e., other-benefiting but self- defecting redivision) after exhibiting prosocial behavior. Study 2 aimed to determine whether rewarding participants economically or morally as compensation would succeed in rebalancing material and moral needs, thereby leading participants to perform the continuously prosocial behavior (i.e., rejecting the unfair and self-benefiting division).

## Study 2

### Method

#### Participants

A total of 28 graduate and undergraduate students in Study 2 came from Liaoning Normal University in China (15 men). The mean age of participants was 22.9 ± 2.0 years old. All participants did not engage in Study 1. The study was approved by the local research ethics committee of Liaoning Normal University.

#### Design and Materials

A 3 (total amount: ¥100, ¥1,000 and ¥10,000) × 4 (division scheme: 9:1, 8:2, 7:3 and 6:4) × 3 (condition: risk, moral reward, and economic reward) within-subject design was used. The acceptance rate for division schemes was used as dependent variable.

**FIGURE 6:**
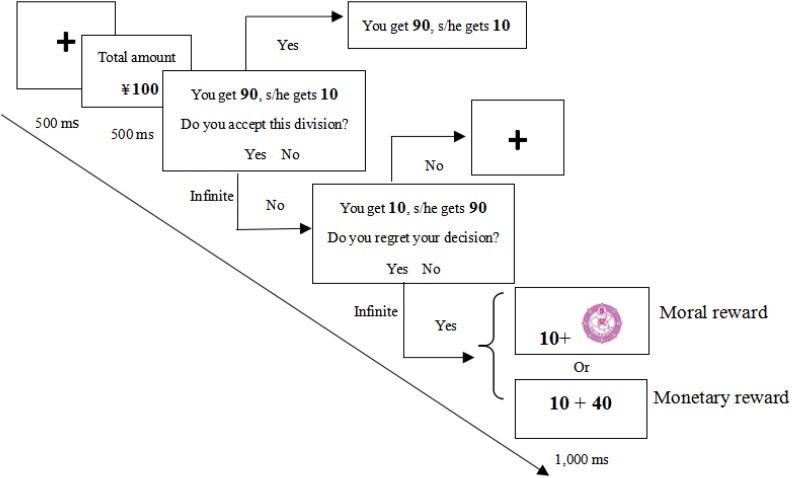
Trial procedure in the moral and economic reward conditions.

Each participant completed three blocks (conditions) of the game. The first block was the same as the risk block in Study 1. The second and third blocks were similar to the risk block, except that participants were required to indicate whether they regretted the preceding choice that followed the fairness but led to the economic loss. If participants regretted their decision, then they would receive compensation with moral or economic reward. Otherwise, the trial was over (**Figure 6**). If a participant did not regret the decision (i.e., rejecting the self-benefiting division), then we inferred that he (or she) may not be concerned about the economic loss in the current trial, and thus, compensation would not be provided. The presentation of the second or third block was counterbalanced across participants. The moral reward was a pictorial medal indexing praise and honor. The economic reward was the additional monetary reward provided by the experimenter. The sum of redistributed money and the economic reward were about 50% of the total remuneration presented at the beginning of a trial. Each of the amount and division schemes was presented 10 times. Thus, each block had 120 trials.

#### Procedure

The procedure in the risk condition was the same as Study 1. Each trial in reward conditions began with a fixation cross, followed by the presentation of a total amount of remuneration to be distributed. Thereafter, a division scheme was presented, whereas two options (“Yes” or “No”) remained visible. If participants accepted the decision, then money was distributed accordingly. Otherwise, redivision was displayed, and participants indicated whether they regretted the preceding decision. If they responded “yes,” then participants would receive a moral or economic reward. If they responded “no,” the trial was over.

### Results

**FIGURE 7:**
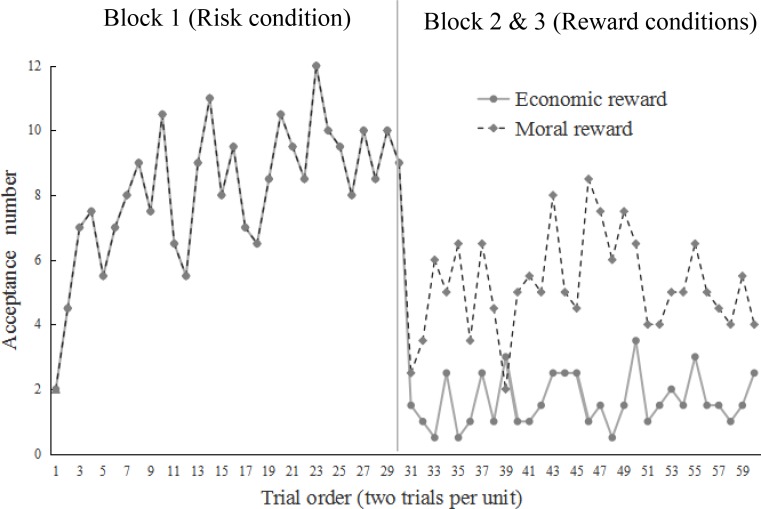
Change of acceptance on extremely self-benefiting division schemes (9:1 and 8:2) in different conditions.

The preliminary test did not indicate order effect (i.e., whether moral reward first or monetary reward first), and the data met the assumptions for ANOVA. The result showed that the acceptance rate on extremely self-benefiting divisions (9:1 and 8:2) in the first two trials was rather low, which was identical to that of Study 1 (**Figure 7**). More participants in the risk condition began to accept division schemes that were self-benefiting but unfair to counterparts when they realized their material resources would be lost after following moral pursuits. However, in the subsequent reward blocks (conditions), participants frequently performed prosocial behavior.

A repeated-measures ANOVA was performed on acceptance rate using total amount, division scheme, and condition as within-subject factors. Results showed that the main effect of condition was significant, *F*(2,54) = 67.23, *p* < 0.001, η^2^ = 0.71, β = 0.11. The mean acceptance rates in the moral (48.7%) and economic reward (29%) conditions were significantly lower than that observed in the risk condition (55%). A significant difference was observed between these two rewarding conditions (*p* < 0.001). In addition, a significant main effect of division scheme was observed, *F*(3,81) = 145.36, *p* < 0.001, η^2^ = 0.84, β = 0.05. Acceptance rates increased significantly as the division scheme approached fairness (5:5). There was no effect of total amount, *F*(2,54) = 0.14, *p* > 0.05, η^2^ = 0.01.

**FIGURE 8:**
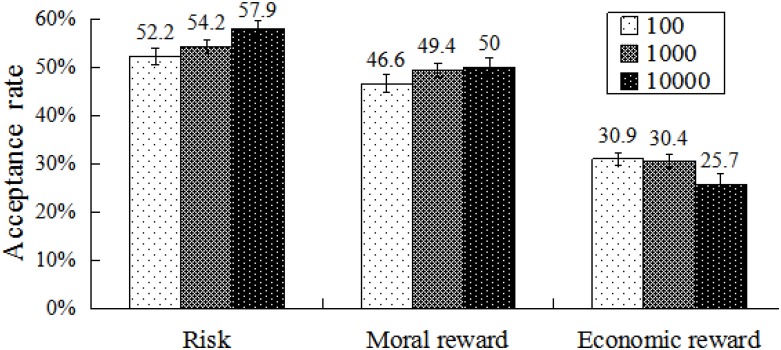
Acceptance rates at different conditions and amounts. Error bars index SE.

There was an interaction between condition and total amount, *F*(4,108) = 4.44, *p* < 0.01, η^2^ = 0.14 (**Figure 8**). Further analyses revealed that acceptance rates differed significantly between any two conditions for all total amounts (all *p*s < 0.01). Acceptance rates did not differ within each pair of total amounts in risk and moral reward conditions. By contrast, acceptance rates of ¥10,000 was significantly lower than that of ¥100 or ¥1000 (all *p*s < 0.05) in the economic reward condition.

**FIGURE 9:**
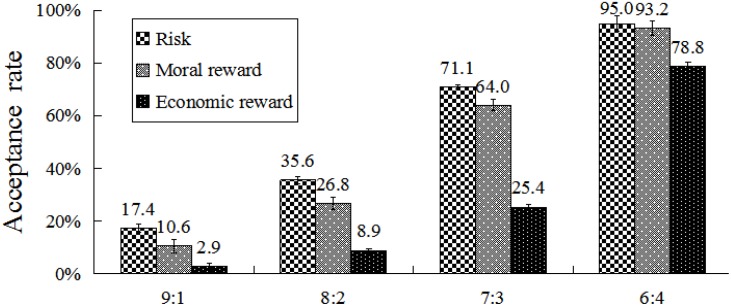
Acceptance rates at different division schemes and conditions. Error bars index SE.

A significant interaction was observed between condition and division scheme, *F*(6,162) = 13.48, *p* < 0.001, η^2^ = 0.33 (**Figure 9**). Further analysis revealed that acceptance rates differed significantly within any pair of division schemes in risk and moral reward conditions (all *p*s < 0.01). However, in the economic reward condition, acceptance rates differed significantly between each pair of division schemes (all *p*s < 0.01), except for the pair of 9:1 and 8:2. For extremely self-benefiting division schemes (9:1 and 8:2), acceptance rates differed significantly between any two conditions (**Table 1**, all *p*s < 0.01). For moderately self-benefiting division schemes (7:3 and 6:4), acceptance rates in the economic reward condition were significantly lower than in other two conditions (all *p*s < 0.001). By contrast, a non-significant difference was found between risk and moral reward condition.

**Table 1:** The results (*p*-value) of pairwise comparisons between different conditions in Study 2.

	9:1	8:2	7:3	6:4
Risk vs. Moral	0.002	0.003	0.092	0.203
Risk vs. Economic	0.000	0.000	0.000	0.006
Moral vs. Economic	0.001	0.001	0.001	0.008


### Discussion

The results of Study 2 showed that acceptance rates of division schemes in the risk, moral, and economic reward conditions decreased gradually, regardless of the total amount of money available. For extremely self-benefiting division schemes (9:1 and 8:2), acceptance rates in moral and economic reward conditions were significantly lower relative to that observed in the risk condition. Hence, moral and economic rewards would compensate for economic loss following social norms compliance. Thus, participants are motivated to reach an internal balance between material and moral needs and to have an internal desire to perform prosocial decision continuously. However, for moderately self-benefiting division schemes (7:3 and 6:4), the acceptance rates for risk and moral reward conditions did not differ significantly. Thus, the compensatory function of morality was less salient relative to that of economic rewards when the material difference between self and others was not obvious.

The effect of moral rewards was modulated by the division scheme. The acceptance rates for moral reward and no-reward (risk) conditions for moderately self-benefiting division schemes (7:3 and 6:4) did not differ significantly. Participants did not consider that the choice of acceptance would absolutely violate fairness norms. Their sense of unfairness was low that the use of moral rewards to promote prosocial behavior was inefficient. However, for extremely self-benefiting division schemes (9:1 and 8:2), acceptance rate in the moral reward condition was significantly lower than that observed in the non-reward condition. Thus, the moral reward effectively compensated for the heavy loss of earned material resources after social norms compliance only if the division scheme was extremely unfair to the counterpart. In addition, moral reward promoted people to regain internal balance between material and moral needs.

In comparison with moral reward, the effect of the economic reward was more salient. Although reward could improve prosocial behavior (Wang et al., 2014), the functions of different types of rewards were not exactly the same (Parks, 2000; Balliet and Van Lange, 2014).

## General Discussion

Fairness norms prevail even in economic situations involving anonymous counterparts; these norms often imply a preference for equal distribution of resources (Radke et al., 2012; Yu et al., 2015). Study 1 demonstrated that social norms compliance was dependent on the outcome of the initial prosocial behavior. By contrast, Study 2 showed that rewards, even moral praise, would compensate for the economic loss following compliance and make participants maintain prosocial behavior. The two studies emphasized the importance of internal, fairness-based balance between material and moral needs in social norm compliance.

### Why Violate Social Norms?

Several studies have demonstrated that most individuals behave with fairness-based prosocial motivations (Radke et al., 2012; Yu et al., 2015). However, many people actually deviate from social norms and perform the unethical behavior, such as dishonesty, deception, cheating, stealing, sabotaging, or breaking the law (Feldman et al., 2015). Traditional economic models (Lewicki, 1984; Smith, 1999) suggested that most people behave according to the prediction of a fully rational, selfish agent who is habitual, automatic, and often operating without conscious thought (Fehr and Gächter, 2000; De Dreu and Nauta, 2009). According to economic models, the participants in our study should accept any self-benefiting division, even though the division scheme is unfair to their counterparts. While we did not reach this result, we found that the participants were still concerned if their economic interests were impaired during the whole experiment. Once they found that the ultimate benefit was reduced, then they would change their behaviors to protect their interests. Behavioral change even includes forgoing of moral needs. Thus, individuals tend to maintain their economic interests when in conflict with moral pursuits.

The psychological need for economic benefits and the moral image is important to everyone. Each individual should not only pursue material benefits as much as possible to meet the need of survival and life, but he (or she) should also consider the moral image. If one person does things that violate social norms to pursue material interests, then his (or her) moral image will be destroyed. Mazar et al. (2008) suggested that people are often torn between two competing motivations of gaining from cheating versus maintaining their positive self-concept as honest individuals.

Therefore, we assumed that the balance of material and moral needs in the coordination between id and superego is important in the formation of prosocial behavior. When the outcome of the initial action is positive or at least neutral – that is, when participants’ ultimate material resources were not reduced after moral pursuits – then, the balance between material and moral needs will be maintained. People will continuously comply with the social norms. Otherwise, they will deviate from social norms to preserve their interests. A modified UG paradigm in which participants responded to a dilemma was used in Study 1. Study 1 showed that most participants rejected extremely self-benefiting division schemes in the first two trials. However, they noted that their ultimate economic income would be reduced in subsequent trials if they continuously pursue fairness. Thus, they then forgo prosocial choice and accept self-beneficial division schemes that are unfair to others.

The finding of Study 1 is consistent with cognitive dissonance theory, which posits that people wish to keep their behavior and belief consistent. When the external incentive is absent, individuals act in accordance with their internal motivation. However, when present, an external incentive can serve as salient behavioral justification and displace norm-based behavior (Festinger, 1957). Economic loss following compliance with social norms could cause cognitive dissonance between “meeting moral rules” and “experiencing material loss.” To deal with cognitive dissonance, participants might regain their balance by exhibiting unethical behavior.

The results our study also supported the integrative model of social value orientation (Van Lange, 1999), which demonstrated that most prosocial individuals continue to perform prosocial behavior until the interdependent others fail to exhibit such a behavior. That is, prosocial individuals become non-cooperative when other individuals do not cooperate. For example, in a task, each participant was required to imagine that he/she had four yellow objects while the other had four blue objects. Each object owned was valued at 50 cents to the person himself/herself and was valued at 100 cents to the other. Participants were paired with several others and were informed that some of these participants decided to give away one, two, or three objects. Participants must decide how many objects they own to give to the other. The results indicated that high cooperative partners induced greater cooperation compared with low-cooperative partners. That is, whether individuals continue to exhibit prosocial behavior is determined by other people or other environmental factors in social interaction. When individuals found they were surrounded by a person or group who showed no cooperation or unfair behavior, then they were less likely to continue exhibiting the prosocial behavior because such behavior could directly lead to the detriment of their interest.

### How to Maintain the Social Norms?

Previous studies consistently showed that sanctions for defection would increase cooperation or other prosocial behavior (Fehr and Gächter, 2002; Nagin and Pogarsky, 2003; Houser et al., 2008; Mazar et al., 2008). However, we argued that the importance of sanctioning defection dissipates when it is used to punish the violator who deviated from social norms because his or her earned material resources have been damaged following repeated moral pursuits. In the risk condition, participants’ initial choice to reject self-benefiting division schemes that are unfair to counterparts might be attributed to reciprocal fairness and prosocial preferences. However, as time progressed, they gradually realized that their earned material resources would be reduced remarkably if they continued to meet moral pursuits. Hence, they had no choice but to deviate from social norms. In this situation, sanctions might be ineffective because of lack of faith in fairness and justice. Over time, an individual would form a stereotypical view in which earned material resources would be lost regardless of compliance with social norms. Other researchers posited that imposing sanctions could be construed as a sign of distrust or create a hostile atmosphere, which would reduce cooperation and cause destructive acts of vengeance (Houser et al., 2008). However, these studies did not demonstrate a method for maintaining people’s prosocial tendencies to voluntary compliance with social norms when sanctions failed. Study 1 indicated that positive outcome on initial behavior (i.e., fair redivision of the money) is the premise of conformity to social norms. Hence, economic or moral rewards were used to compensate for economic loss following conformity in Study 2. The results showed that social and monetary incentives were effective in providing compensation to internal fairness. This finding strongly indicated that when the individual suffered a heavy loss of earned material resources after performing prosocial deed (e.g., being cheated after a donation), providing him or her with rewards, even purely moral praise, could effectively help him or her to regain internal balance between material and moral needs.

This study was the first to explore the compensatory function of moral rewards in the social domain. The effectiveness of moral rewards in promoting human prosocial behavior might be explained by individual desire to establish a positive social image, which has been suggested as an important factor in promoting human cooperation (Grimalda et al., 2016). Building a positive social image is an automatic and more efficient means to enforce cooperation because other individuals might preferentially cooperate with those who have a good reputation (Ghang and Nowak, 2015). Therefore, a forward-looking and rational agent prefers to choose moral cooperation for future benefits and focus on establishing a positive social image. Although the compensatory function of moral rewards was relatively lower than that of economic rewards, moral rewards effectively improved prosocial behavior.

The role of moral reward in maintaining social norms is consistent with the theory of self-concept maintenance (Mazar and Ariely, 2006). This theory indicated that when people attend to their moral standards, any dishonest action is likely to be reflected in their self-concept (they will update their self-concept as a consequence of their actions), which, in turn, will cause people to adhere to the strict delineation of honest and dishonest behavior. Thus, increasing attention to internal honesty standards would decrease the tendency for dishonesty. In an experiment, participants were first asked to write down either the names of 10 books they had read in high school (no moral reminder) or the Ten Commandments (moral reminder). The Ten Commandments pertain to moral rules and are expected to increase attention to participants’ own moral standards (Shariff and Norenzayan, 2007). Each participant then received a numerical test, which consisted of 20 matrices. Each matrix is based on a set of 12 three-digit numbers. Participants were required to find two numbers that added up to 10 in a limited time (4 min). Finally, participants either had their answer to the experimenter (control condition) or orally indicated the number of correctly solved tests (a condition in which participants have a chance to cheat). The results indicated that the participants who wrote the book names are more likely to cheat when given the opportunity. However, the participants who wrote down the Ten Commandments did not. This study shows that prosocial behavior increases when a person is concerned with moral information. The results of our study (Study 2) also demonstrated that participants showed more attention to their positive image and maintain their self-concept when provided with moral pedals.

Hence, prosocial tendencies of reciprocal fairness are not lasting when people repeatedly comply with social norms but experience earned mate loss. The only means to maintain their prosocial tendencies when facing this situation is through immediate compensation via social (morality) or economic (money) incentives and by reaching a balance between material and moral needs.

### Limitations and Future Directions

The two studies were conducted in a laboratory setting and involved money priming. Although we found that the amount of money influenced fair consideration to a certain degree, the level of money priming might have been low for participants. For example, the acceptance rate should increase alongside the amount of total money in the risk condition. A larger amount of total money leads to higher loss of earned material resources following rejection. By contrast, in the non-risk condition, the acceptance rate should decrease alongside the amount of total money because a larger amount of total money leads to higher guilt among participants upon acceptance. However, our results were not consistent with our assumption. This result might be due to the participants experiencing difficulty in imagining experimental money as real money, which was presented as a number. Future research would benefit from investigating this effect via different methodological approaches, such as virtual or natural environment to improve ecological validity (Winking and Mizer, 2013; Patil et al., 2017). Another limitation is the fact that the participant’s economic condition could affect results. Fairness and justice are concerned with three principles, namely, equity, equality, and need; however, UG just emphasizes the value of equality (Deutsch, 1975). Therefore, other important areas for future studies include participants’ need for money. The third limitation is about the fact that a control condition, such as an equal division scheme (e.g., 5:5), was not included. Using the control condition might help us investigate if individuals are indeed driven by equality/prosocial values, rather than merely monetary gains. The last limitation is that the sample size seems rather small (40 participants for Study 1 and 28 participants for Study 2), even though all follow the within-participant design, and the statistical tests were significant with a sufficient power. Further study with a larger sample is needed to verify the findings of the present study.

## Author Contributions

FL conceived and designed the experiments. XW and JH performed the experiments and analyzed the data. JH, FL, and BC wrote the paper.

## Conflict of Interest Statement

The authors declare that the research was conducted in the absence of any commercial or financial relationships that could be construed as a potential conflict of interest.

## Footnotes

1 The proposer and the responder fully understand the mechanism for the evolution of cooperation specificity of the experimental context and the manipulations.

2 The number of acceptance are calculated for every two trials. For example, if four participants accept the division at the 11th trial and six participants accept the division at the 12th trial, then the number of acceptance for the 11–12th trials is 10.

## Appendix 1

**Table A1:** The amount of money in re-division for each condition.

Total division			100¥	1,000¥	10,000¥
9/1	Accept		90	900	9000
	Reject	No-risk	40/50/60	400/500/600	4000/5000/6000
		Risk	40/50/60/20/10	400/500/600/200/100	4000/5000/6000/2000/1000
8/2	Accept		80	800	8000
	Reject	No-risk	40/50/60	400/500/600	4000/5000/6000
		Risk	40/50/60/20/10	400/500/600/200/100	4 000/5000/6000/2000/1000
7/3	Accept		70	700	7000
	Reject	No-risk	40/50/60	400/500/600	4000/5000/6000
		Risk	40/50/60/20/10	400/500/600/200/100	4000/5000/6000/2000/1000
6/4	Accept		60	600	6000
	Reject	No-risk	40/50	400/500	4000/5000
		Risk	40/50/60/20/10	400/500/600/200/100	4000/5000/6000/2000/1000

*Each participant performed two conditions (blocks) of the game. In the non-risk block, if participants rejected the random division that is self-benefiting but unfair for the counterpart, they always received a positive outcome (i.e., a fair redivision). That is, they would get 40, 50, or 60% of the total remuneration. In the risk block, if participants rejected the offer, they possibly received a negative outcome. That is, in the redivision they might merely get 10 or 20% of the total remuneration.*
